# Comparative Analysis of Micrometer-Sized Particle Deposition in the Olfactory Regions of Adult and Pediatric Nasal Cavities: A Computational Study

**DOI:** 10.3390/pharmaceutics16060722

**Published:** 2024-05-27

**Authors:** Ziyu Jin, Gang Guo, Aibing Yu, Hua Qian, Zhenbo Tong

**Affiliations:** 1 School of Energy and Environment, Southeast University, Nanjing 210096, China; zjin0038@student.monash.edu (Z.J.); guogang55@163.com (G.G.); 2 Southeast University—Monash University Joint Research Institute, Suzhou 215123, China; aibing.yu@monash.edu; 3 ARC Hub for Computational Particle Technology, Department of Chemical Engineering, Monash University, Clayton, VIC 3800, Australia

**Keywords:** nose-to-brain drug delivery, olfactory region, CFD (computational fluid dynamics), micrometer-sized particles, drug targeting

## Abstract

Direct nose-to-brain drug delivery, a promising approach for treating neurological disorders, faces challenges due to anatomical variations between adults and children. This study aims to investigate the spatial particle deposition of micron-sized particles in the nasal cavity among adult and pediatric subjects. This study focuses on the olfactory region considering the effect of intrasubject parameters and particle properties. Two child and two adult nose models were developed based on computed tomography (CT) images, in which the olfactory region of the four nasal cavity models comprises 7% to 10% of the total nasal cavity area. Computational Fluid Dynamics (CFD) coupled with a discrete phase model (DPM) was implemented to simulate the particle transport and deposition. To study the deposition of micrometer-sized drugs in the human nasal cavity during a seated posture, particles with diameters ranging from 1 to 100 μm were considered under a flow rate of 15 LPM. The nasal cavity area of adults is approximately 1.2 to 2 times larger than that of children. The results show that the regional deposition fraction of the olfactory region in all subjects was meager for 1–100 µm particles, with the highest deposition fraction of 5.7%. The deposition fraction of the whole nasal cavity increased with the increasing particle size. Crucially, we identified a correlation between regional deposition distribution and nasal cavity geometry, offering valuable insights for optimizing intranasal drug delivery.

## 1. Introduction

In nasal drug delivery research, the focus is on delivering drugs directly to the brain via the olfactory region, bypassing the blood–brain barrier (BBB) through intranasal administration. This approach leverages three pathways: olfactory, trigeminal nerve transmission, and cellular transport, utilizing the nasal cavity’s structure to transport drugs directly to the brain, avoiding the BBB. The olfactory region connects directly to the brain’s olfactory bulb, making it a unique conduit for drug delivery, offering advantages like non-invasiveness and rapid effect [1]. De Lorenzo et al. made a conclusive discovery of inhaled gold nanoparticles translocating to the brain in squirrel monkeys [2]. Sequential images captured in experiments depict the entire process from nanoparticles depositing on the olfactory mucosa to their entry into the olfactory bulb and subsequent arrival in the brain. Although direct evidence of the olfactory pathway has been primarily demonstrated in animal subjects [3,4], there is increasing evidence that the nasal olfactory route is a viable pathway for human brain accumulation of particles [5]. Hence, nasal-to-brain administration has been proposed for treating various central nervous system diseases, such as migraines [2], sleep disorders [6], and Alzheimer’s disease [7].

The nasal cavity serves as the first line of defense against particulate pollutants like dust and viruses [8], and apart from respiration, it also efficiently regulates the temperature and humidity of incoming air [9]. The geometric configuration of the nasal airways is intricate, and the interior of the human nasal cavity is notably delicate and susceptible, complicating the direct measurement of airflow velocity and particulate deposition within in vivo settings. Consequently, researchers resort to Computational Fluid Dynamics (CFD) for conducting simulation-based investigations [10,11]. Since the early 1990s, Computational Fluid Dynamics (CFD) has been utilized for simulating and analyzing airflow distribution within the nasal cavity. Early efforts, grounded in coronal CAT scan imagery, successfully created three-dimensional geometric models of the nasal cavity of healthy adults, offering an initial understanding of airflow distribution through the nasal passages [12]. As development has progressed, model construction techniques have diversified, including three-dimensional models of the nasal cavities of healthy adults and infants based on CT scans [13,14]. The advancement of these methodologies enhances the design accuracy of intranasal drug delivery systems and offers a swift, economical tool for evaluating devices like nasal sprays, thereby significantly improving the assessment efficiency of airflow and particulate deposition impacts within the nasal cavity.

This study employs microparticles based on their unique therapeutic and technological advantages due to their structural and functional properties. Their benefits include targeted delivery, degradation protection, minimized systemic side effects, precise dosing, uniform distribution, predictable pharmacokinetics, and reduced variability [15]. The latest research on intranasal brain-targeted micrometer-sized drug particles includes rivastigmine microspheres for treating Alzheimer’s disease [16] and chitosan-coated lipid microparticles for nasal administration of resveratrol [17].

Current devices for nasal inhalation medication for infants mainly rely on designs intended for adults, and during tidal breathing, children experience greater deposition of medication in the upper respiratory tract [18,19]. Therefore, research into the deposition of particles in adult and child nasal cavities is needed to improve the dosage and methods suitable for children. Morphological and size differences exist between adult and child nasal cavities, with many in vivo and in vitro studies considering the deposition of micron-sized particles in adult nasal airways [20] and some focusing on children’s nasal deposition [14]. Research on olfactory region particle deposition has mainly concentrated on studies of nanometer-sized particle deposition in adults [21,22].

The primary goal of this study is to investigate the deposition patterns of drug particles in the nasal cavities of adults and children through simulations, particularly focusing on the differences in deposition within the olfactory region. By analyzing these deposition patterns, this study aims to improve and evaluate the susceptibility of children to particle exposure and to optimize drug dosages and administration methods. This is crucial for enhancing the effectiveness of drugs delivered to the olfactory region.

## 2. Methodology

### 2.1. Airway Model and Mesh

**Figure 1:**
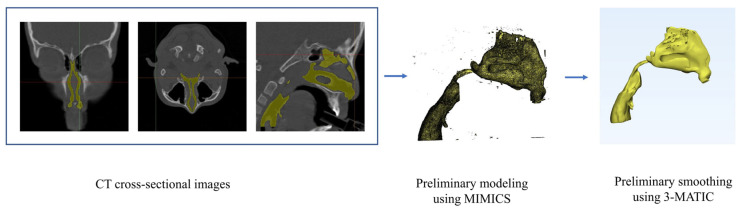
Flowchart from CT data to 3D nasal cavity model construction for numerical simulation.

In this study, nasal cavity models of a 9-year-old child (male, 36 kg), a 10-year-old child (female, 32 kg), a 31-year-old adult (female, 61 kg), and a 59-year-old adult (male, 75 kg) were selected, all of whom were in a healthy nasal condition. The computed tomography (CT) scans used in this study were obtained from imaging and radiology departments, with ethical approval granted for the research. Detailed information from the CT scans was anonymized for privacy concerns. The chosen nasal cavities were free of any nasal deformities or respiratory-related issues, making them suitable for simulating nasal airflow and studying particle deposition patterns within the nasal cavity. The slice increments for the four models were 0.5 mm, 0.5 mm, 1 mm, and 1 mm, respectively, adequate for generating computational models of the nasal cavity, with all four models having a pixel resolution of 512 × 512. The development of the nasal cavity models employed MIMICS 21.0 image processing software, which allows for the segmentation of required parts from imported CT scans based on defined thresholds. In this study, the thresholds were set between −1024 HU and −212 HU (HU—Hounsfield Units), values chosen based on previous research conducted by the researchers [23]. The software facilitated the visualization of segmented nasal cavity models, which were further refined and developed slice by slice to achieve satisfactory nasal cavity models. To preserve the originality of the respiratory structures, they were exported into another software named 3-MATIC 13.0 for only minor smoothing operations to avoid compromising the anatomical integrity of the nasal cavity. The procedural steps for the establishment of the model are depicted Figure 1.

**Figure 2:**
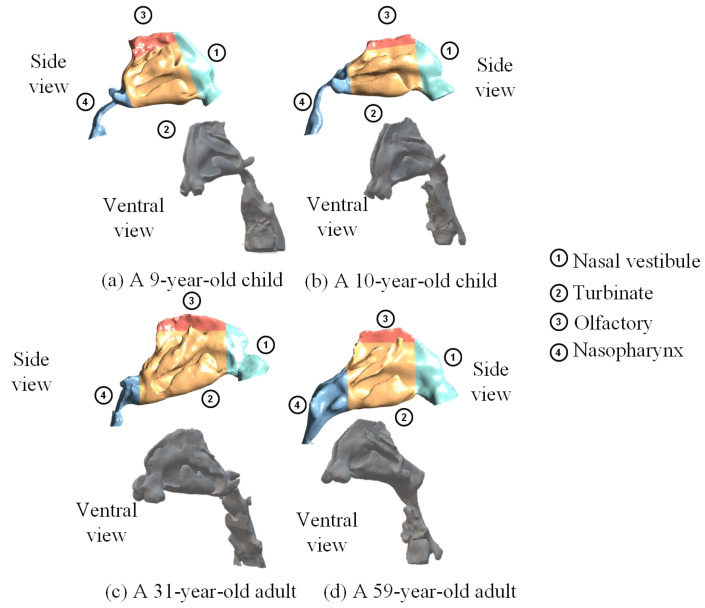
Modeling and segmentation of the nasal cavity model.

For the quantitative assessment of the flow field and particle deposition, surface generation and model partitioning were conducted using SpaceClaim. The nasal cavity model was divided into four distinct parts, namely the olfactory region, nasal vestibule, central nasal passage, and nasopharynx [24], and the specific segmentation of each model is illustrated in Figure 2. The total nasal area, airway volume, olfactory area, the percentage of the olfactory area to the total area, and the hydraulic diameters at the inlet and outlet are provided. The calculation of the total nasal area includes the region from the nostrils to the end of the pharynx. The data highlight the geometric structural differences in the distribution of the entire nasal cavity and the olfactory region among human subjects. The delineation of the olfactory region is primarily considered the part of the nasal passage covered by the olfactory epithelium. Currently, the olfactory cells are mainly identified and distinguished histologically through their unique cellular composition and structure [25,26]. Imaging studies suggest that techniques such as Single Photon Emission Computed Tomography (SPECT)/X-ray CT and Magnetic Resonance Imaging (MRI) can be used, employing mutual information methods to fuse SPECT and MRI images, to visualize the location of the olfactory epithelium in vivo, assessing the thallium transport pathway from the nasal cavity to the anterior cranial olfactory bulb [27]. The most accurate location and extent of the olfactory epithelium can be obtained from continuous histological sections of nasal specimens [28]. In this paper, the area of the olfactory region accounts for 7–8% of the total area, aligning with the modeling requirements outlined in the literature [21]. The detailed geometric data regarding the nasal cavity model is illustrated in Table 1.

**Figure 3:**
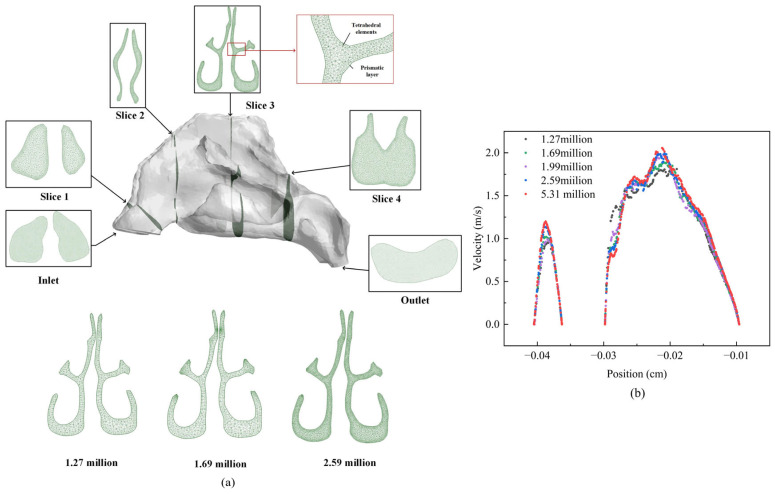
(**a**) Illustration of the nasal cavity grid; (**b**) validation of grid independence.

The nasal cavity, ranging from the nostrils to the pharyngeal section, was selected as the subject for study, and the grid was partitioned accordingly. Fluent mesh was used to create an unstructured mesh due to the complex structure of the nasal cavity. To enhance computational accuracy in the olfactory region, a denser grid was chosen for this area. To ensure accuracy in the nasal cavity boundary layer, five layers of boundary were set on the nasal cavity wall surface. A schematic diagram of the grid cross-sections is shown in Figure 3.

For a more precise numerical analysis of the limited number of grids divided in the nasal cavity, it is necessary to optimize the grid design. Conducting a grid independence verification can ensure the reliability of the simulation results. Therefore, a grid independence verification was carried out before the simulation. Five grids were delineated with respective grid counts of 1.27 million (coarse grid), 1.69 million (relatively coarse grid), 1.99 million, 2.59 million, and 5.31 million (fine grid). The pressure differences at the inlet and outlet were set at −40 Pa, and the velocities at geometrically identical positions were analyzed and compared under these five grid numbers. The verification results showed that when the grid number changed from 1.69 million to 1.99 million, the variation rate was greater than 20%, while from 1.99 million to 2.59 million, the variation rate was less than 2%. Using 2.59 million grids was sufficient to meet the accuracy requirements while using smaller computational resources. The validation of grid independence is shown in Figure 3.

### 2.2. CFD Model

#### 2.2.1. Modeling

This paper employed the Computational Fluid Dynamics (CFD)-based software tool Fluent 2020 R1 to solve the airflow control equations within the intricate structure of the human nasal cavity. Disregarding periodic conditions, the flow field was assumed to be constant to highlight the influence of particle shape on the locations where deposition occurs. A steady flow rate of 15 LPM, which mimics the typical breathing pattern during rest and while sitting, was simulated. This flow was assumed to be smooth and non-turbulent, also known as laminar flow. The airflow within the nasal cavity was presumed to be constant, unable to be compressed, and characterized by smoothness. The equations that govern the airflow are as follows:

(1) $$\begin{array}{cc} \mathrm{Continuity}: & \;\;\;\;\;\;\;\;\;\frac{\partial u_{i}}{\partial x_{i}}=0 \end{array}$$

(2) $$\begin{array}{cc} \mathrm{Momentum}: & \;\;\;\;u_{j}\frac{\partial u_{i}}{\partial x_{j}}=-\frac{1}{\rho }\frac{\partial p}{\partial x_{i}}+v\frac{\partial^{2}u_{i}}{\partial x_{j}\partial x_{j}} \end{array}$$

where $u_{i}$ is the air velocity in three Cartesian coordinate directions, i.e., *i* = 1, 2, and 3, *p* is the pressure, *ρ* is the fluid density, v is the air kinematic viscosity, *j* is an index for spatial dimensions, and $x_{j}$ and $x_{i}$ are spatial coordinates.

The computation of the transportation and deposition of micron-sized particles used the discrete phase model (DPM) [29], describing particle motion through the Lagrangian approach. This model tracks individual particles in the flow field and is suitable for calculations involving small particles. The particle force balance equation is given as follows:

(3) $$m_{p}\frac{d{\vec{u}}_{p}}{dt}=\vec{F_{D}}+\vec{F_{G}}+\vec{F}$$

where $m_{p}$ is the particle mass, ${\vec{u}}_{p}$ is the particle velocity vector, $\vec{F_{D}}$ is the drag force, $\vec{F_{G}}$ is the gravity force, and $\vec{F}$ is the additional force. The drag force is expressed as follows:

(4) $$\vec{F_{D}}=m_{p}\frac{\left(\vec{u_{f}}-\vec{u_{p}}\right)}{f}$$

The drag force per unit particle mass *F_D_* is defined as

(5) $$F_{D}=\frac{18\mu_{g}}{\rho_{p}d_{p}^{2}}\frac{c_{D}{Re}_{p}}{24}$$

where *d_p_* is the particle diameter and *μ_g_* is the fluid dynamic viscosity, $\rho_{p}$ is the particle density, and Re*_p_* is the particle Reynolds number. 

*C_D_* is the drag coefficient, which can be obtained using the following equation, where $a_{1}$, $a_{2}$, $a_{3}$ are coefficients [30]:

(6) $$C_{D}=a_{1}+\frac{a_{2}}{Re}+\frac{a_{3}}{Re^{2}}$$

Re*_p_* is the particle Reynolds number given by

(7) $$Re=\frac{\rho D_{p}\left|\vec{u_{p}}-\vec{u_{f}}\right|}{\mu }\;$$

where ρ is the fluid density, $D_{p}$ is the particle diameter, and *μ* is the fluid dynamic viscosity.

The Navier–Stokes equations and the continuity equation are solved using the FLUENT’s commercial CFD code to provide aerodynamic data such as pressure, velocity, streamlines, and wall shear stress. The process of spatial discretization is carried out via the second-order upwind scheme. The pressure-velocity coupling is resolved with the SIMPLE approach. Throughout all computations performed, it was assumed that the airflow was both incompressible and stable. The intake plane is positioned close to the nostrils, while the outlet is located beneath the nasopharynx. A static respiratory flow rate of 15 LPM was used to specify a uniform velocity that is perpendicular to the outflow plane. This flow rate passes across the whole nasal cavity. 

#### 2.2.2. Boundary Conditions and Computational Parameters

A flow rate of 15 LPM was used to simulate the average breathing rate during a calm state. The Reynolds number at the model’s outlet is 994; therefore, a laminar flow model is considered for calculations. The airway walls are presumed to conform to a no-slip condition, and the influence of gravity on airflow cannot be neglected. The minimal concentration of particles in the airflow resulted in the disregard of the influence of particle motion on the flow. Therefore, the first phase consisted of simulating the airflow field, which was then followed by calculating the trajectories of individual particles. The Lagrangian Particle Tracking method was utilized to track particles that were released at the nasal input, assuming that all particles were spherical in shape. The simulated air density for the airflow was 1.1845 kg/m^3^, whereas the dynamic viscosity was 1.84 × $10^{-5}$ kg/m·s. The enforced boundary conditions included no-slip conditions at the walls of the nasal airway, zero pressure at the entrance of the nostril, and negative pressure at the outflow. A substantial number of particles were simulated to ensure that the results were not biased by the quantity of particles emitted. The simulation encompassed aerodynamic particle sizes spanning from 5 to 100 µm, with distinct sizes of 5, 8, 10, 12, 15, 18, 20, 30, 50, 80, and 100 µm. In the particle deposition simulations, primarily single particle size studies were conducted, with an input of fifty thousand particles per simulation, and the particle density was 1000 kg/m^3^. Considering that the particles are in a dilute phase, interactions between particles were not taken into account [29,31]. The DPM boundary condition was set to “escape” at the inlet and outlet, while “trap” was prescribed at the walls [32].

### 2.3. Data Processing

The term “deposition fraction” refers to the ratio of the number of particles deposited in the entire nasal passage or a specific region to the total number of particles released from the nostrils. The formula for calculating the deposition fraction in the nasal cavity is as follows:

$$Deposition\;Fraction\;=\;\frac{Number\;of\;particle\;trapped\;by\;the\;part}{Number\;of\;particles\;injected\;from\;nostril\;inlets}$$

Deposition efficiency refers to the ratio of the number of particles deposited in a given area to the number of particles that enter that area.

$$Deposition\;Efficience\;=\;\frac{Number\;of\;particle\;trapped\;by\;the\;part}{Number\;of\;particles\;injected\;in\;this\;part}$$

## 3. Model Validation

To verify the reliability of the simulation results of the computational model, an inertial parameter (IP) was used, defined as IP = ${da}^{2}$Q, where da is the particle aerodynamic diameter (μm) and Q is the flow rate (LPM) [33]. The data obtained from simulations were compared with existing literature data to confirm the reliability of the computational model simulation.

**Figure 4:**
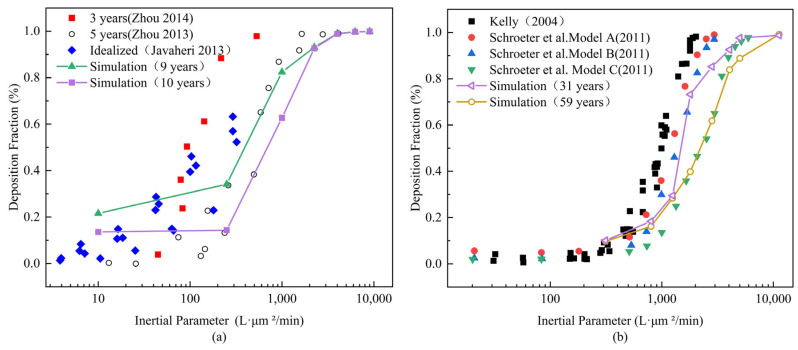
Validation of nasal deposition in child and adult: (**a**) Validation of the child nasal cavity model and (**b**) Validation of the adult nasal cavity model [20,21,34,38,39].

To verify the accuracy of our model in capturing the involved dynamics, the simulation results for the adults aged 31 and 59 were compared with the in vitro experimental data of Kelly et al. and Schroeter [34,35,36]. Kelly used a replica model of a Caucasian adult male nasal cavity scan, while Schroeter et al.’s CFD simulation utilized models with three different surface roughness levels. The deposition fraction data derived from the simulations displayed an S-shaped inertial deposition curve and demonstrated good consistency with other experimental data, especially the nasal cavity model of the 31-year-old adult closely matching Schroeter’s Model B curve, and the 59-year-old adult nasal cavity simulation closely aligning with Schroeter’s Model C curve. Similarly, the simulation results for the children aged 9 and 10 were compared with prior literature, also showing good consistency and closely aligning with the outcomes of 5-year experiments [37,38,39]. For the children, when the inertial parameter IP < 300 L·${\mu m}^{2}$/min, the deposition rate remains <20%. As the inertial parameter IP increases, the deposition fraction sharply increases, reaching complete deposition when IP > 3000 L·${\mu m}^{2}$/min. For the adults, when the inertial parameter IP < 1000 L·${\mu m}^{2}$/min, the deposition rate remains < 20%. As the inertial parameter IP increases, the deposition fraction increases sharply, reaching complete deposition when IP > 10,000 L·${\mu m}^{2}$/min. Thus, both the adult and child models allow for a direct comparison of simulation and measured deposition results. The comparative results of the child and adult models are shown in Figure 4. 

**Figure 5:**
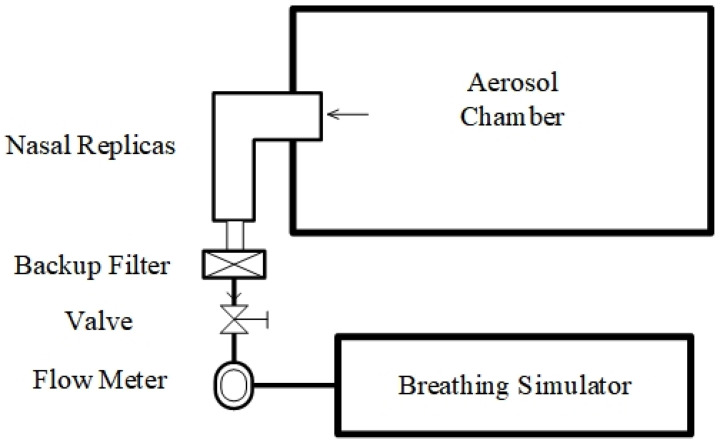
Schematic diagram of in vitro aerosol deposition setup.

**Figure 6:**
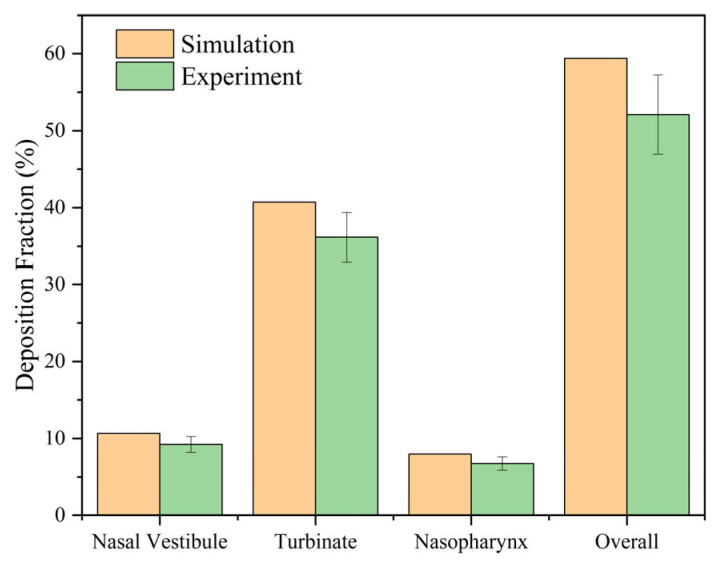
Comparison results between simulation and experiment.

Additionally, an in vitro deposition experiment was conducted to validate the simulation results. The experimental setup is depicted in Figure 5. A 3D-printed nasal cast was utilized to emulate the real nasal cavity, and an ultraviolet-visible spectrophotometer (UV-1800, Mapada, Shanghai, China) was employed to quantify the deposition within specific regions, while the human respiratory state was simulated using a breathing simulator (HRH-BRM-6600, Huironghe, Beijing, China). The use of the UV spectrophotometer includes the following steps: (1) prepare the sample; (2) calibrate the spectrophotometer; (3) measure the sample; (4) analyze the data. The 3D printing of the nasal cavity model utilized resin material, and a coating was applied to the surface to simulate mucus. The experimental procedure has been delineated in our prior work [40]. The experimental outcomes and the corresponding simulation results at an aerosol flow rate of 15 LPM with a particle diameter of 10 μm are illustrated in Figure 6. The comparison of the results between the simulation and experiment demonstrate the reliability of the simulation. The in vitro experiments employed a model of a 31-year-old adult for simulation validation.

## 4. Result

### 4.1. Airway Parameter

There were significant morphological and size differences in the nasal airways among the four subjects (a 9-year-old child, a 10-year-old child, a 31-year-old adult, and a 59-year-old adult). In terms of nasal cavity morphology, children exhibited shorter overall nasal cavity lengths, elongated nasal vestibules, and thinner pharyngeal walls, with similarities in cross-sectional area changes across various regions compared to adults. With age, the size and complexity of the lower and middle nasal passages increased.

**Figure 7:**
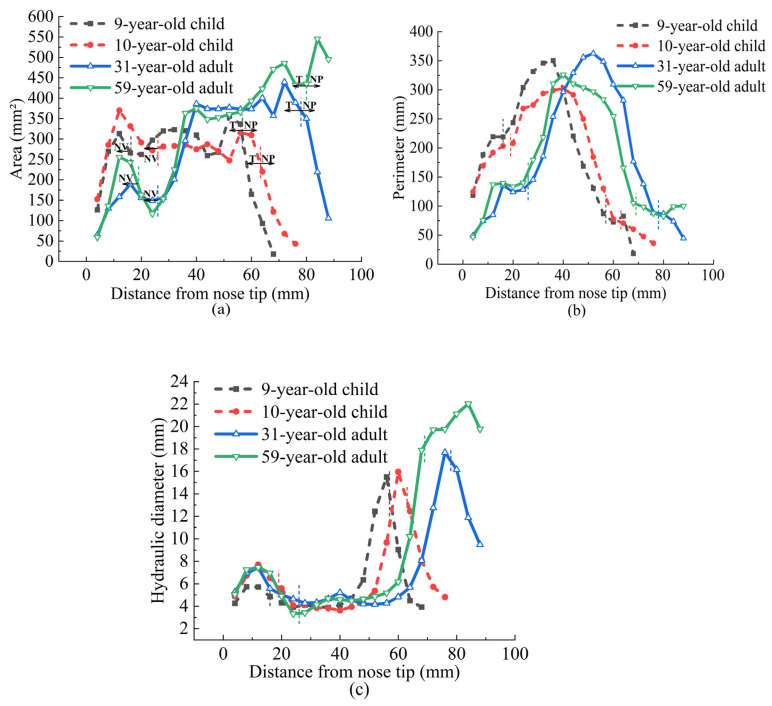
Comparison of geometric dimensions on the cross-section from nostril tip between adults and children: (**a**) area dimensions; (**b**) perimeter dimensions; (**c**) hydraulic diameter.

Graphical representations detailed the size comparison of the nasal airways of the four subjects, with the horizontal axis representing the function of distance from the nostril tip. Figure 7 illustrates the comparative analysis of the nasal cavity’s cross-sectional area, cross-sectional perimeter, and hydraulic diameter at each segment. In terms of nasal cavity length, the 9-year-old child’s and 10-year-old child’s nasal cavities were 75.42% and 78.36% of the length of the 31-year-old adult’s and 85.73% and 89.07% of the 59-year-old adult’s, respectively. These data indicate that nasal cavity development in children is not yet complete, with shorter lengths, but the length does not necessarily increase with age after adulthood. Considering the overall nasal airway volume, the 9-year-old child’s and 10-year-old child’s nasal areas were 56.70% and 56.83% of the 31-year-old adult’s and 65.86% and 74.65% of the 59-year-old adult’s, respectively. In the cross-sectional area, the adults had larger areas than the children in the posterior region of the middle nasal passage, with a more gradual change in the children’s cross-sectional area. Notably, both the children and adults displayed similar trends in cross-sectional area changes in the same geometrical regions. The maximum perimeter in the children was closer to the nostril tip. As shown in Figure 7, the hydraulic diameters at the front end of the nasal cavity were very similar between the adults and children, with the maximum hydraulic diameter in the children closer to the front end. When considering the olfactory region, children have a smaller olfactory area than adults, but the proportion of the olfactory area is similar. Moreover, compared to adults, the standard deviation of the cross-sectional area within the olfactory segment is smaller in children.

In summary, the adults had longer nasal cavities than children, with larger cross-sectional areas in the latter half of the nasal conchae. The children’s nasal cavity perimeters and hydraulic diameters peaked near the nostril tip. Both the adults and children showed consistent trends in changes in cross-sectional area, perimeter, and hydraulic diameter. These data are crucial for understanding drug transport and deposition in different age groups during respiration. 

### 4.2. Flow Pattern

**Figure 8:**
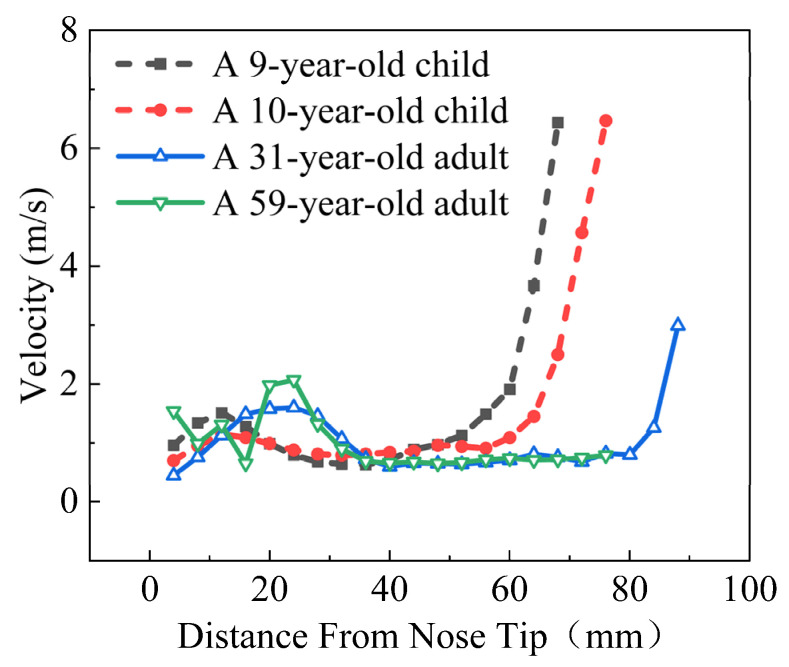
Velocity distribution across the cross-sectional area in adults and children.

This section discusses the airway velocity distribution from the nostril entrance to the nasopharynx in both adults and children. Average velocities were measured every 4 mm of the nostril tip. It was observed that the children’s average sectional velocities were slightly higher than the adults’, possibly due to children’s smaller cross-sectional areas of nasal cavities. Pediatric subjects demonstrate a subdued velocity distribution between 20 and 60 mm from the nostril tip, contrasting with adults who exhibit a similarly gradual distribution between 40 and 80 mm. Additionally, both adult and pediatric populations exhibit a restrained modulation in the cross-sectional area within this comparable range, implying that regions characterized by minimal alterations in the nasal cavity internal area correspond to restricted variations in velocity. The velocity distribution within the nasal cavity is illustrated in Figure 8.

**Figure 9:**
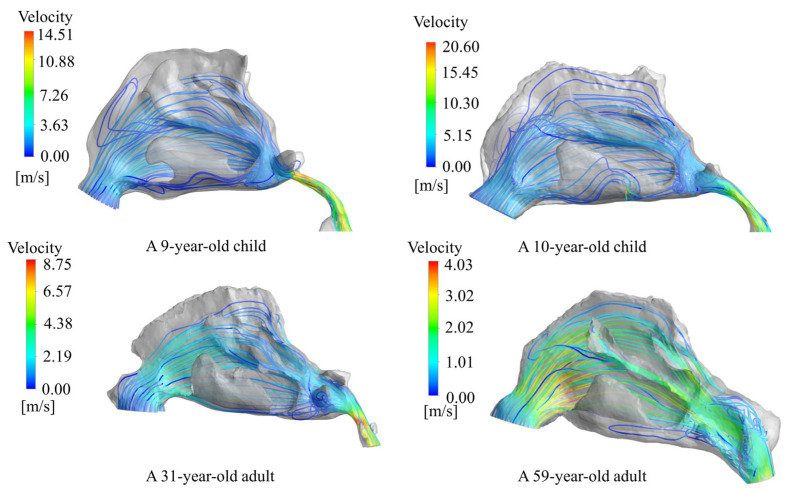
Distribution of nasal cavity velocity streamlines in children and adults.

The airflow distribution within the nasal cavity is depicted in Figure 9. Airflow enters the nasal vestibule evenly through the nostrils, then experiences a 90° change in direction caused by centrifugal forces, which mostly directs the flow towards the upper nasal cavity. The majority of the airflow traverses the central nasal channel, while a small quantity flows through the upper nasal passage and olfactory region, before making a 90° turn upon entering the nasopharynx. The highest velocities are attained at the most constricted cross-section. Airflow exerts a profound influence on particle deposition within the nasal cavity. An examination of streamline diagrams for the four nasal cavities revealed that both the adults and children experience peak velocities predominantly in the pharynx, with the children exhibiting slightly elevated maximum sectional velocities compared to the adults. In the nasal cavity, particularly in the upper half proximal to the olfactory region, the streamlines demonstrate an almost horizontal orientation parallel to the olfactory area. Particle deposition mechanisms typically involve inertial collision, Brownian motion, and gravitational settling. In the olfactory region, the nearly parallel airflow may diminish particle deposition resulting from inertial collision, given that particles are less likely to directly impact the walls of the olfactory area. Specialized strategies may be requisite to augment drug deposition in the olfactory region, encompassing interventions such as adjusting particle size, employing auxiliary devices to modify aerodynamic characteristics, or utilizing specific drug formulations.

### 4.3. Deposition Pattern

**Figure 10:**
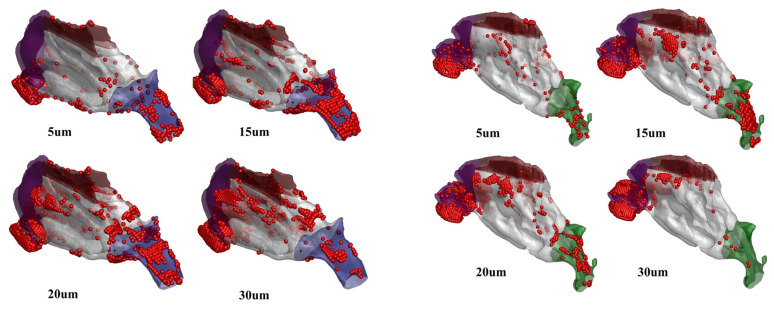
Schematic illustration of the deposition distribution of particles of different sizes in the nasal cavities of a 10-year-old child and a 31-year-old adult.

Figure 10 illustrates that particle deposition predominantly occurs in the lower part of the nasal vestibule near the nostril tip, with larger particles tending to be more abundant in the nasal vestibule. The deposition patterns in the left and right nasal cavities of humans demonstrate asymmetry. In both the children and the adults, particles of approximately 5 μm are uniformly distributed in this region, while particles in the range of 15–30 μm tend to concentrate more in the upper part of the middle vestibule.

#### 4.3.1. Comparison of Deposition Fractions

**Figure 11:**
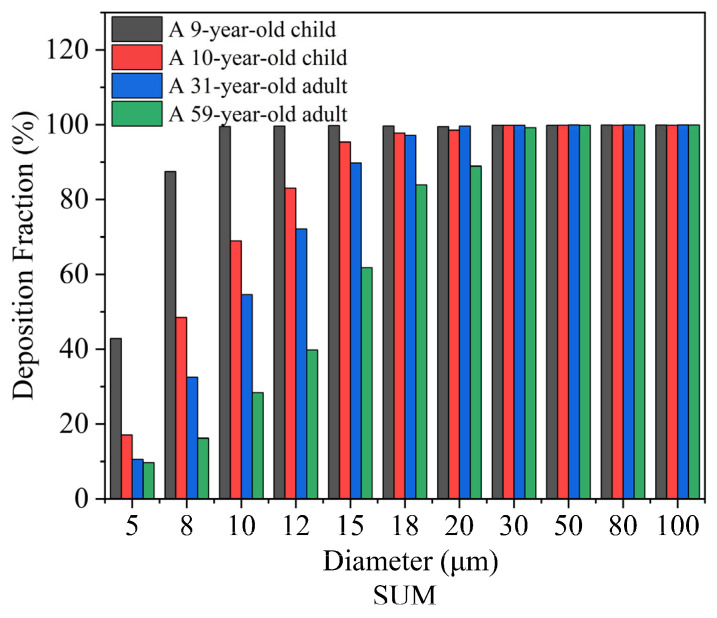
Total nasal deposition fraction in adults and children at a flow rate of 15 LPM.

Considering overall nasal cavity deposition at a flow rate of 15 LPM, this study discussed the deposition of 5–100 micrometer particles in the nasal cavity. Figure 11 illustrates that as the particle diameter increases, the overall nasal cavity deposition increases, reaching almost complete deposition in both the children and adults when the inhaled particle diameter exceeds 30 μm. At a particle diameter of 5 μm, the percentage of inhaled particles deposited in the nasal cavity of the children was 42.8% and 17%, while in the adults, it was 10.5% and 9.75%. It was observed that at small particle diameters of 5–12 μm, the children had slightly higher particle deposition in the nasal passages than the adults. This is primarily due to the narrower airways in the nasal cavities of children compared to adults, resulting in higher airflow velocity in children’s airways at the same flow rate, leading to greater particle deposition on the nasal mucosal surface. It can be concluded that at 5–20 μm, total nasal cavity deposition in the children was higher than in the adults, and after 30 μm, the overall deposition areas in both the children and adults were consistent. 

**Figure 12:**
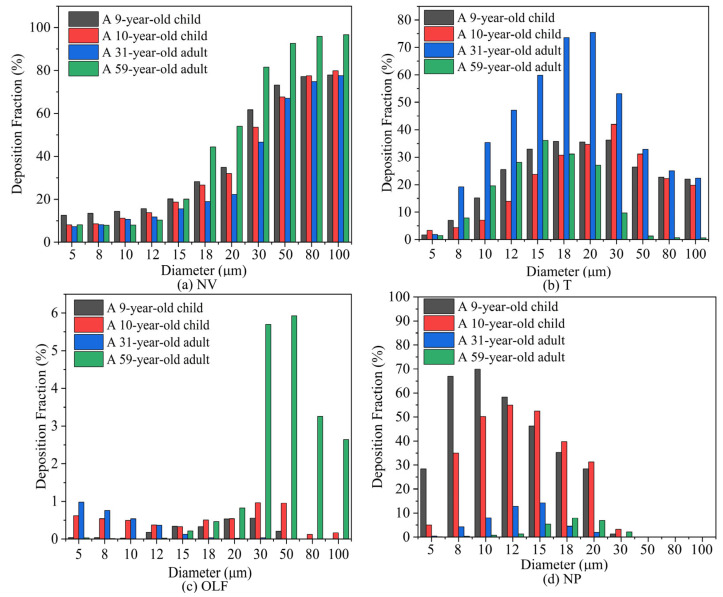
Deposition fraction in various regions for adults and children at a flow rate of 15 LPM: (**a**) nasal vestibule; (**b**) turbinate; (**c**) olfactory region; (**d**) nasopharynx.

Figure 12 shows the variation in the deposition fraction in different nasal regions with particle size. Figure 12a illustrates the impact of particle diameter on the nasal vestibule (NV), indicating that the deposition fraction (DF) gradually increases with the increase in particle size. Figure 12b presents the effect of particle size on the turbinate, revealing that the deposition fraction in the turbinate first increases and then decreases with increasing particle size. Figure 12c examines the impact of particle diameter on olfactory region delivery, showing that the delivery efficiency to the olfactory region is generally low. This is mainly because, on the one hand, the increase in particle size enhances particle inertia, increasing the probability of collision with the wall. On the other hand, the increase in particle size leads to increased deposition in the nasal valve area, reducing the number of particles entering the turbinate, resulting in the observed trend of first increasing and then decreasing. Figure 12d shows the impact of particle diameter on nasopharyngeal deposition, indicating that the deposition fraction in the nasopharynx initially increases and then decreases with the increase in particle size.

When considering regional particle deposition, in the nasal vestibule, it was seen that with increasing particle diameter, deposition gradually increased in both the children and adults. At 5 μm, nasal vestibule deposition in the children was about 12.6% and 8.1%, while in the adults, it was around 7.2% and 8.1%. At 5–15 μm, the deposition fractions in the nasal vestibule were similar between the children and adults, though slightly higher in the children. At 30 μm, the adults’ maximum deposition rate reached 81.5%, while the children’s particle deposition was above 50%. When considering larger-diameter micrometer particles for nasal cavity deposition, over half would deposit in the nasal vestibule.

As the particle diameter increases in the turbinate region, it can be observed that both the adults and children exhibit a trend of increasing and then decreasing nasal particle deposition fractions with the growing particle size. For the 9-year-old child, the deposition fraction reaches its maximum at a particle diameter of 18 μm, while for the 10-year-old child, it peaks at 30 μm. The 31-year-old adult shows the highest deposition at 20 μm, and the 59-year-old adult exhibits the maximum deposition at 15 μm.

In the olfactory region, situated at the superior aspect of the nasal cavity, the airflow velocity approaches near-zero levels upon reaching the apex, thereby resulting in a relatively diminished total particle count deposited in the olfactory region. At a flow rate of 15 LPM, olfactory deposition was generally below 1%, with the 59-year-old adult male showing a significant increase at 30 μm, with olfactory deposition reaching 5.9%. At a particle diameter of 15 μm, both the adults and children showed deposition in the nasal olfactory area.

#### 4.3.2. Particle Deposition Efficiency

**Figure 13:**
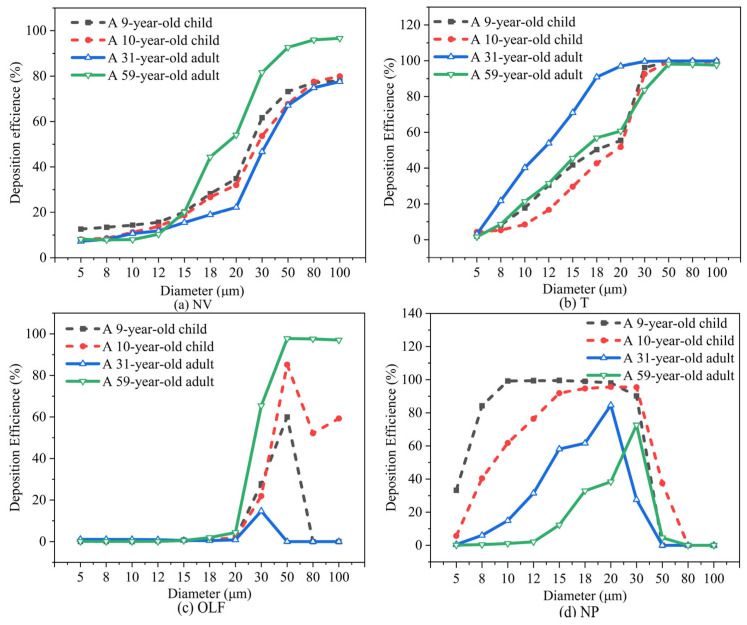
Variation in deposition efficiency in different nasal regions with particle size in adults and children: (**a**) nasal vestibule; (**b**) turbinate; (**c**) olfactory region; (**d**) nasopharynx.

Figure 13 illustrates the variation in deposition efficiency across different regions of the nasal cavity for the adults and children as a function of particle size. Deposition efficiency in the nasal vestibule and turbinate areas of both the adults and children increased with particle diameter. The adults exhibited a marginally higher deposition efficiency in the turbinate area compared to the children. In the nasopharyngeal area, the children’s deposition efficiency was slightly higher than the adults. With increasing particle diameter, the deposition efficiency gradually increased in the smaller diameter range and, as particle diameter continued to increase, most particles deposited at the front end of the nasal airway, resulting in low deposition efficiency in the nasopharyngeal area for larger particles. At a flow rate of 15 LPM, the particle deposition efficiency in the nasal vestibule and turbinate regions increases with the diameter of micrometer-sized particles for both the adults and children. The nasal cavity models of the 9-year-old and 10-year-old children, exhibiting similar ages, demonstrate consistent trends in interception efficiency across various regions. This suggests that age can be considered a relevant factor when assessing particle deposition in nasal airways. 

## 5. Discussion

This investigation conducted numerical analyses on airflow and particle deposition within the nasal airways of realistic human child and adult models, derived from CT scans. It unveiled marked discrepancies in airflow dynamics and deposition patterns across varying age groups, employing models from two children and two adults to highlight the anatomical distinctions in nasopharyngeal structures. These disparities were manifested not only in the dimensions but also in the morphology of the airways, with the adults displaying regions of cross-sectional areas up to twice those of the children and significantly greater lengths and volumes.

This study elucidates how intranasal structural variations across age groups impact airflow characteristics and, consequently, the transport and deposition sites of therapeutic particles. Such insights are crucial for designing optimized drug delivery systems that ensure targeted medication delivery. At a constant airflow rate of 15 LPM, the children’s nasal passages exhibited slightly higher airflow velocities at the posterior end than the adults. Airflow streamline patterns and particle deposition outcomes suggest substantial inter-age-group differences, emphasizing the insufficiency of adult-focused deposition studies for accurately prescribing pediatric dosages.

Particle characteristics, including size, shape, and density, alongside nasal cavity geometry, dictate deposition within the nasal cavity. This study found significant variations in particle distribution between the adults and children, with noteworthy findings on olfactory area deposition under steady breathing conditions. Specifically, it demonstrated the potential for adjusting particle size to enhance targeted deposition within the olfactory region, shedding light on the challenges of direct olfactory deposition from nasal inhalation without specialized equipment.

Furthermore, this research underscores the absence of age-specific correlations in nasal cavity deposition, advocating for detailed consideration of age due to its strong linkage with nasopharyngeal anatomy and size. Despite limitations such as steady-flow assumptions and idealized conditions, this study underscores the necessity to expand nasal cavity deposition research to include children and the elderly with respiratory conditions. Addressing these special populations will illuminate individual variances, facilitating more effective medication formulation and delivery strategies across different age groups. 

## 6. Conclusions

This study conducted a detailed comparative analysis of micrometer-sized particle deposition in realistic nasal cavity models of children and adults from the perspective of fluid particle dynamics. It systematically elaborated on the differences in nasal cavity geometry, flow field, and deposition between children and adults. The results indicate that adults have a larger olfactory area compared to children, yet the ratio of the olfactory area to the total nasal cavity area is similar between the two groups. In both the children and adults, the airflow streamlines near the olfactory region are almost parallel to the olfactory area and have lower velocities. At a particle diameter of 15 μm, particle deposition was observed in the olfactory regions of both the adults and children. Suppose that medication is inhaled directly through the nostrils. In that case, it is challenging for the drug to reach the olfactory region, necessitating specialized equipment to assist, reduce the interception of drug particles by the anterior turbinate, and allow the particles to pass through the anterior turbinate to reach the middle nasal passage. Due to individual variability, future research will require the analysis of a larger number of subjects to promote more effective medication formulation and delivery strategies across different age groups.

## Figures and Tables

**Table 1:** Nasal airway dimensions in airway models of four different age groups.

Model	Olfactory Area (mm^2^)	Olfactory Volume (mm^3^)	Whole Area (mm^2^)	Olfactory Region Area Relative to the Total Area (%)	Whole Volume (mm^3^)	$D_{in}$	$D_{out}$
A 9-year-old child	1325	914	15,472	8.56	18,738	2.42	2.56
A 10-year-old child	1379	793	15,951	8.65	18,780	1.94	2.57
A 31-year-old adult	1567	1087	20,687	7.57	33,047.11	2.36	2.93
A 59-year-old adult	1496	841	18,699	8.00	28,453.34	2.81	2.38

$D_{in}$ is the equivalent diameter of the nostrils at the inlet (mm); $D_{out}$ is the equivalent diameter of the nasopharynx at the outlet (mm).

## Data Availability

The original contributions presented in the study are included in the article; further inquiries can be directed to the corresponding authors.
